# NEWS

**DOI:** 10.7189/jogh.06.010203

**Published:** 2016-06

## DEMOGRAPHY

➢ In 2003, China relaxed its restriction on divorce, and divorce rates have risen rapidly–a remarkable transformation in a country where marriage was universal and permanent. This reflects the underlying transformation of Chinese society, with mass urban migration dividing couples, the improving status of women, and increased prosperity making it more feasible to live alone. Divorce is easier and cheaper in China compared to most other countries, and divorce rates are approaching levels in the USA. Often, falling divorce rates are a reflection of falling marriage rates and rising births outside wedlock, but neither hold true in China where marriage rates, including remarriage, remain high. Marital tensions can be worsened by the pressure on young Chinese people to marry early – and they often lack opportunities to meet suitable potential partners, and cohabitation before marriage is still rare (although increasing). Moreover, although women initiate over 50% of divorces, settlements still tend to favour men. (*The Economist*, 23 Jan 2016)

➢ According to the UN Population Fund, 25% of the world’s population is youthful (aged 10–24 years), with the vast majority living in the developing world. In India, home to the world’s largest number of young, working–age people, 1 million people reach 18 years every month–and at 422 million, the number of people aged 15–34 years is equivalent to the combined populations of the USA, Canada and the UK. This is partly due to global successes in reducing child mortality, and more children being enrolled in school. This generation of young people is more likely to be educated, connected and ambitious – leading them to more mobile. Globally, the youth bulge is felt unevenly – in Germany, the median age is over 46 years, compared to 18 years in Nigeria. Governments face the staggering challenge of job creation to absorb these new workers – 40% of whom are either unemployed or working in insecure jobs at poverty pay. Youth unemployment is a strong predictor for social unrest, and to combat the uneven generation gap, countries with older populations need more migration from younger countries, alongside job creation in the global south. (*New York Times*, 5 Mar 2016)

➢ The transformation of Medellin, Colombia’s capital city, has been recognized by its winning the Lee Kuan Yew World City Prize, which is awarded by the Singapore government. This award honors outstanding urban achievements and solutions, and the Nominating Committee praised Medellin’s transformation from uncontrolled urban expansion and violence, to its status as model for urban innovation. The committee made special mention of the political will, leadership, and long–term plans shown by the city’s past three mayors, which have tackled security problems, aided economic development and improved its citizens’ quality of life and employability. Medellin’s current mayor, Federico Guitierez Zuluaga said, “this is an important recognition that we feel proud of for our city. We thank you for the encouragement to continue working for our city, a spectacular city that has come a long way but also has a long way to go.” (*Cities Today*, 18 Mar 2016)

➢ Japan’s extreme demographic challenges follow on from 20 difficult years for the country, which has been beset with deflation, budget deficits and high public debt. The first challenge is Japan’s rapidly shrinking and aging population, with the share of people aged over 65 years rising from 5 in 1950 to 25% in 2012 – the highest figure in the world, and its median age is 45.9 years, compared to the global median of 29 years, and 38.7 years for other OECD countries. This trend is set to continue, while the country’s population is expected to decrease by 22–23%. This is compounded by Japan’s low fertility rates, partly as long working hours and high population density in urban areas discourage women from having children. However, Japan’s overseas–born population was 1.7%, significantly below other OECD countries, so migration is an untapped potential for population growth. Also, carefully managing longevity gains (eg, healthier aging, longer careers, efficient health care) can help offset the economic impacts of aging. (*OECD*, 11 Apr 2016)

➢ Life expectancy continues to increase in the UK, and men’s life expectancy is slowly catching up with women’s. However, underlying these very welcome developments is a new, and worrying trend – for the first time since the 19^th^ century, the narrowing of the gap in life expectancy between rich and poor people has reversed. Increases in life expectancy before 1940 were due to clean water, improved sanitation, affordable housing and cleaner air, among other things–and this benefitted poorer people as wealthier people could afford healthier environments and lifestyles. Post–1950, advances in health care tended to benefit all sections of society equally, so the life–expectancy gap remained constant. However, this began to change in the 1990s, mainly due to lifestyle factors (eg, diet, exercise, alcohol and tobacco use) which increase the risk of non–communicable diseases. For example, previously smoking was evenly spread throughout all social groups, and 82% of UK men smoked in 1948. This has now fallen to 21%, and poorer men are more likely to smoke. The rich – poor divide in unhealthy lifestyles is widening the life – expectancy gap, but tackling individual lifestyles may be more difficult than wider public health measures like cleaning up water supplies. (*New Scientist*, 3 May 2016)

## ECONOMY

➢ Small states such as Botswana, the Seychelles and Mauritius rank the highest among African states in most human development indices. This is highly beneficial to their own citizens in terms of health, prosperity, safety and good governance, but has little impact beyond their own borders. States which have larger impact – eg, Nigeria, Kenya and South Africa – do not fare so well. If Kenya’s economic dynamism translated into GDP levels equivalent to Mauritius, oil–rich Nigeria’s governance matched Botswana’s, and South Africa’s post–apartheid moral stature continued to endure, this could have profound impact on their neighbors and Africa in terms of development, peace and security. Although each country falls short of its own potential, some indicators (eg, Nigeria’s free elections in 2014, Kenya’s entrepreneurism and South Africa’s international standing) hint at their potential to lift the entire continent. Improving linkages between these countries and their neighbors – politically, economically and culturally–is vital to ensuring the success of the pan–African Union, and the East African Community (Africa’s most integrated regional bloc) showcases the benefits of integration. (*dailymaverick.co.za*, 22 Jan 2016)

➢ Underneath the public health concerns raised over the zika virus as it spreads among 26 countries in the Americas, lies a quieter question of economic loss and hardship caused by the virus. While it is too early to give a definitive answer on the potential financial havoc wrecked by the illness, estimates can be gleaned from the impact of another illness spread by the Aedes mosquito–dengue fever. Donald Shepard of Brandeis University estimates that the 2013 outbreak of dengue fever cost the global economy US$ 8.9 billion, with the largest burden shouldered by developing countries. This does not include the impact on tourism, and already shares in travel companies are falling as pregnant women and those planning pregnancies are advised against visiting affected countries. This will hit Brazil especially hard, as it hosts its annual Carnival festivities and anticipates 500 000 tourists for the August 2016 Olympic Games. (*Bloomberg View*, 5 Feb 2016)

➢ The International Monetary Fund (IMF) has signaled that it will downgrade its outlook for the global economy in April. This follows recent warnings from the OECD that global economic growth will slow within the next few months. However, there are few signs that governments are heeding these warnings – indeed, a senior US Treasury official commented that it is not reasonable to expect a crisis response over economic uncertainty. The IMF is concerned that any announcement of joint action by the G20 group of leading economies could risk the global economic expansion. Part of the problem is a shortage of tools to deal with the next economic crisis, and the IMF wants the G20 to boost spending, delay interest rate rises and for the European Central Bank to boost stimulus efforts. (*Wall Street Journal*, 8 Mar 2016)

➢ Mr John Mangudya, the governor of Zimbabwe’s central bank, confirmed that the country expects its first International Monetary Fund (IMF) loan since 1999 later in 2016, after paying off foreign lenders. The exact amount has yet to be agreed, but the IMF has agreed to double the amount available to Zimbabwe to US$ 984 million. Zimbabwe is trying to emerge from international isolation, which are largely blamed on its government’s policies. Its worst drought since 1994 has left 4 million Zimbabweans facing hunger, and forced the government to lower its economic growth forecast from 2.7% to below 2% – the IMF and World Bank forecast growth of 1.4% and 1.5% respectively. As part of the loan agreement, the government agreed to major reforms, including compensation for evicted farmers and a reduction in public sector wages. Once Zimbabwe’s arrears are cleared, it will be ready for rating by international ratings agencies with the aim of issuing bonds. (*Reuters*, 16 Mar 2016)

➢ China’s foreign capital reserves fell by US$ 28.57 billion in February, marking the fourth consecutive monthly decline, albeit at a decelerating rate. The government is examining a range of measures to curb speculative foreign transactions. These measures include a levy designed to penalise short–term currency speculation (the so–called “Tobin tax”), and imposing fees on the sales of forward positions. “We are considering policies to increase the costs of short–term speculation as long as they don’t affect normal capital flows,” says Mr Wang Yungui, the head of regulation at China’s State Administration of Foreign Exchange. (*WSJ*, 22 Mar 2016)

## ENERGY

➢ 1.1 billion people – mainly in southern Asia and sub–Saharan Africa – lack electricity, and electrification is barely keeping pace with population growth in the latter. Some entrepreneurs are using technology to provide access to clean, cheap systems which are metered and paid for by mobile technology. The user by–passes electricity grids, instead harvesting solar energy from rooftop panels that are connected to batteries that store energy until nightfall. This is more suitable for rural dwellers with low energy needs and high grid connection costs. Urban areas could benefit from pre–paid meters topped up by mobile phone – and higher revenues would encourage more investment. There is tension between countries electrifying using cheaper fossil fuels and risking severe pollution problems akin to Beijing and New Delhi, against clean energy that may be more expensive and intermittent. However, regional transmission networks that share power, alongside “baseload” energy systems (geothermal, hydro, natural gas) that operate constantly can support business needs, while smaller–scale solar power to cover households’ energy needs could form part of the solution. (*The Economist*, 27 Feb 2016)

➢ According to the news agency, Xinhua, China’s gas consumption rose by 3.5% in 2015–the smallest increase in a decade, and lower than the official 5.7% forecast. Gas for domestic usage was the largest component (69%, or 131.8 billion m^3^) of overall consumption. This is the second year of sluggish growth in gas consumption (partly caused by weaker economic growth), despite government efforts to promote gas over coal as a cleaner energy source. These figures bring China’s gas market to below the International Energy Agency’s long–range growth forecast of 4.7%. The downturn is adding to a glut of gas supplies, with current prices lower than contract prices, and firms are attempting to postpone gas shipments. Australia, which has invested heavily in gas projects in China, has been badly affected by the downturn, leading to Western Australia’s credit rating being downgraded by Moody’s. The Chinese government has attempted to raise demand by slashing prices, but this has deterred domestic production and led to even lower output. (*Radio Free Asia*, 29 Feb 2016)

➢ Since industrialisation 250 years ago, humans have released 500 billion tonnes of CO_2_ into the atmosphere from fossil fuels and deforestation–and is set to release another 500 billion tonnes over the next 40 years. 50% of a CO_2_ increase is removed from the atmosphere within 30 years, 30% is removed within a few centuries, and the remaining 20% may remain for millennia. Carbon capture and storage (CCS)–a technology which captures CO_2_ at emission and stores it underground, eg, in depleted oil and gas reservoirs–could be a solution. The International Energy Agency (IEA) estimate that CCS is the most effective way of reducing CO_2_ emissions by 13% by 2050, but note that progress in CCS implementation is slower than hoped. There is a current wave of 22 CCS projects worldwide, and they will collectively capture 48 million tonnes of CO_2_ each year from coal–fired power stations, gas processing and other industrial processes. However, there are few further CCS projects in the pipeline, and the world risks losing momentum without policy intervention. The IEA believes that the world has more than enough CO_2_ storage resources, but more investment in exploration and development is required. (*OECD*, 20 Apr 2016)

➢ Venezuela has been forced to cut its national power supply for 4 hours daily, to last over the next 40 days – due to reduced rainfall that drives electricity–generating turbines in hydroelectric dams. The cuts will affect 10 out of 23 states, including major cities. This is an additional blow to the country’s citizens, who already face shortages of food and medicine. However, the oil sector is unlikely to be included in the cuts thanks to its importance to the Venezuelan economy. The economy is already struggling with falling oil prices, and is set to shrink by 8% in 2016, and the power shortages will cause further economic damage. (*Newsweek*, 22 Apr 2016)

➢ In an interview shortly before his death, Prof Sir David MacKay–the UK’s former chief scientific adviser–called the idea of renewable energy powering the UK “an appalling delusion.” He believes that solar, wind and biomass energy would require too much land, huge battery back–ups and cost too much to be viable options for the UK, although he believes that solar power has great potential in hot, sunnier countries. He notes that renewable energy – which produces 1% of the UK’s electricity–cannot sufficiently be scaled up. Instead, he calls for the UK to focus on nuclear energy and carbon capture storage technology to reach zero carbon emissions. He believes that carbon capture and storage is essential in tackling climate change, and is disappointed by the UK’s lack of progress with the technology. (*The Guardian*, 3 May 2016)

## ENVIRONMENT

➢ As President Obama’s last term draws to a close, the US Supreme Court put on hold his administration’s Clean Air Act. This Act – seen as a key legacy of Obama’s administration – is designed to reduce emissions from power plants by 32% by 2030, and is the main tool for the USA to meet its emissions targets agreed at December’s UN climate talks. Various business groups and 27 states (led by West Virginia and Texas) launched the bid to block the Act, arguing that it would devastate their economies. The decision means that the regulations will not take effect while court battles continue over their legality, and raises doubts over the long–term future of the Environmental Protection Agency. “We are thrilled that the Supreme Court realised the rule’s immediate impact and froze its implementation, protecting workers and saving countless dollars as our fight against it continues,” said Mr Patrick Morrisey, the Attorney General for West Virginia. (*Scientific American*, 9 Feb 2016)

➢ Mongolia is currently experiencing a *dzud *– a natural disaster which occurs when a summer drought is followed by heavy winter snowfall that makes already scarce pastures inaccessible to livestock. Previously, dzuds occurred once a decade, but they have recently been occurring every few years. It is believed that the increasing frequency could be due to a combination of climate change – the average temperature in Mongolia has risen by 2.1 °C since 1940 and Mongolia is ranked the 8^th^ most vulnerable country to the impact of climate change – and human activity. In a country where 50% of people rely on livestock production, oversupply of animal products has led to falling prices – and increased animal numbers to maintain incomes. When combined with climate change, this has had a devastating effect on Mongolia’s pastoral land, with over 70% being degraded, and increasing forest fires has reduced forest area by 0.46% each year – and threatening Mongolia’s ancient way of life. The Ministry of Foreign Affairs has asked for international help to deal with the dzud – an estimated US$ 4.4 million is needed for emergency vehicles, medicine, food and livestock supplies – but the government has stopped short of declaring a state of emergency. (*IRIN*, 7 Mar 2016)

➢ A study by the Frankfurt School of Finance and Management for the UN Environment Programme shows that a record US$ 286 billion was invested in renewable energy in 2015 – more than double the investment in fossil fuels. In another milestone, developing countries’ investment in renewable energy – US$ 156 billion – outstripped the US$ 130 billion investment by developed countries for the first time. China and India have led the way in developing countries’ investment in renewables, and the USA increased its investment by 19%. However, Europe’s investment in renewables fell by 21% in 2015, despite being a previous trailblazer. These investments are beginning to have an impact on climate change, and the International Energy Agency has pinpointed their growth as the main reason why global CO_2_ emissions have been stable for 2 years, despite 6% economic growth. Without them, annual CO_2_ emissions would be 5% – or 1.5 billion tonnes – higher. There are concerns that in the short–term, low prices for fossil fuels could spark a surge in their use, although pledges made at December’s climate summit in Paris should limit this, and it is not expected to have a lasting impact. (*New Scientist*, 24 Mar 2016)

➢ Hokeng Metal Processing Co.’s industrial plant, located in Nonthong Village in Lao, appears to be pumping polluted waste water into the neighboring area. The plant extracts valuable copper, lead and other valuable minerals from discarded electronics, and re–sells them worldwide. Water contamination is 16 times higher than normal levels. Although Lao’s Ministry for Natural Resources claim they are working on the plant’s pollution management, no formal action has yet been taken against the company. The company may also have failed to comply with international regulations on transferring hazardous materials from developed to less developed countries. (*Radio Free Asia*, 7 Apr 2016)

➢ Cycling and walking are normally healthy activities, but a study published in *Preventative Medicine* found that air pollution in heavily polluted cities – such as Delhi in India, Karachi in Pakistan and Doha in Qatar – means that the harms can outweigh the benefits. In Delhi, cycling is only beneficial if people cycle for less than 5 hours a week, so if people’s daily commute is longer than 30 minutes each way, cycling will damage their health. People who cycle for longer each day, eg, bike couriers, will experience even more harm. Air pollution is strongly linked to heart disease and lung cancer, and causes thousands of death a year – and exercise can intensify its harmful effects as heavy breathing causes more dirty air to be drawn into the lungs. (*New Scientist*, 5 May 2016)

## FOOD, WATER AND SANITATION

➢ Following floods in 2015, the northern state of Arakan in Myanmar has seen a sharp rise in the number of severely and moderately malnourished children. The flooding – caused by heavy rain and Cyclone Komen – has destroyed crops and rice paddies and has contaminated water sources. The numbers of malnourished children aged under 5 years seen by a European Commission food program in Maungdaw district rose to 1500 in October, compared to 500 in July. The children are eating fewer and less diverse foodstuffs, sometimes relying on rice and water only. The real number is likely to be much higher, as the food programmes only see the minority of affected children. 90% of Arakan’s population belong to the Muslim minority Rohingya, who face violence and discrimination with no legal recognition of their citizenship, and in 2015, 14 000 of the state’s children were admitted to this program, including 10 900 children aged under 5 years. (*Irrawaddy*, 28 Jan 2016)

➢ In 2015, Papua New Guinea experienced a severe drought, arising from the El Niño weather system, followed by floods and mudslides after heavy rains in February 2016. According to the UN Office for the Co–ordination of Humanitarian Affairs, 480 000 people are facing critical food shortages and are in need of food aid. The extreme weather conditions has also caused health care facilities to close, or operate at lower capacity due to water shortages or problems with storage. Aid agencies are working with the government to distribute food and monitor dengue outbreaks in Daru, and possible cases in Kiunga. (*Reuters*, 4 Mar 2016)

➢ About 60% of Africa’s farms are less than one hectare, and agriculture employs more than 50% of people in sub–Saharan Africa. Therefore, improving agricultural productivity is one of the best ways to raise Africa’s living standards – and its farms are less productive than Latin American and Asian farms. The value of Africa’s agriculture has increased by 400% since 1961, albeit by bringing more land into cultivation rather than improving yields, so output per person actually fell. There are inherent difficulties in increasing yields due to infertile soils, and varying climate patterns make crops more heterogeneous and less amenable to a “green revolution” – unlike Asia’s staple crops of rice and wheat. Inadequate roads, price controls and corruption over subsidies to poor farmers also hinder agricultural development. However, hybrid seeds are improving yields, the gradual lowering of tariffs encourage exports, and land reforms give more control to women farmers. This gradual brightening is underpinned by better governance and fewer conflicts across the continent. Improved roads, information on market prices, better storage and food processing to aid diversification and job creation, better husbandry to boost livestock production would all further support Africa’s agricultural evolution. (*The Economist*, 12 Mar 2016)

➢ According to the charity Water Aid, India has the world’s highest number of people without access to clean water, with 75.8 billion people – or 5% of India’s population – being forced to buy expensive water or use contaminated supplies. Buying water costs up to US$ 0.72 a day – nearly 20% of a poor person’s income – and diarrhea kills 140 000 children in India each year. India’s water problems are set to worsen, as rivers become more polluted, groundwater reserves diminish, and global warming causes more erratic rainfall. It is predicted that India can only meet 50% of its water needs within 15 years. Indian states are experimenting with measures to improve water supply and management, including privatization, water filtration units and water kiosks in drought–prone areas. Water shortages could cause tensions, and Satya Tripathia, an advocate in India’s Supreme Court says “the government really has to pay attention. Water is the one thing that can tear this country apart.” (*Yahoo*, 22 Mar 2016)

➢ On the first anniversary of the earthquake which struck Nepal on 25 April 2015, thousands of people across the country still face problems of water, sanitation and hygiene. Swift action by the government, community health workers and aid organisations immediately after the earthquake prevented disease outbreak, but more aid and delivery is urgently required to ensure that rebuilding happens as quickly as possible. Basic infrastructure was badly damaged by the earthquake, and natural springs – a major source of water for rural Nepalese people – produce less water, or have dried up completely. Some communities that previously had continuous access to water only have access for 1–2 hours a day, and many people are having to wash in rivers or queue for hours at taps. The aid organization, WaterAid, is working with local partners and communities to find new spring sources and build water supply infrastructure. (*The Himalayan*, 26 Apr 2016)

## PEACE AND HUMAN RIGHTS

➢ Crime rates in Japan are exceptionally low by international standards, and those arrested for minor crimes are treated with leniency – less than 5% of those found guilty of a penal offense are sent to prison. Japan emphasizes rehabilitation, and has extremely low rates of re–offending. However, 99.8% of prosecutions end in a guilty verdict, and the system relies heavily on confessions – ie, 90% of criminal prosecutions. There are few safeguards for suspects being questioned, as they can be held for 23 days without charge, often with little contact with a lawyer. Few interrogations are recorded, and although physical torture is rare other methods such as sleep deprivation are not. Moral blackmail (eg, citing shame brought on family), and fabricating confessions and pressurising the suspect to sign them can happen. Once in court, the non–adversarial nature of the trial system means that judges seldom question whether confessions are voluntary. According to one estimate, 10% of all convictions leading to a prison sentence are based on false confession. There have been recent miscarriages of justice – a mother convicted of murdering her daughter was released after her innocence was proved by a crime reconstruction; and Iwao Hakamada was freed after 46 years on death row after his conviction was declared unsafe – he appears to have been tortured when arrested. (*The Economist*, 5 Dec 2015)

➢ Latin America has consistently been home to 86% of the world’s most violent cities, according to data published since 2011 by the Mexican Citizens’ Council for Public Security – an NGO whose annual survey assesses the world’s 50 most violent cities. It found that not only does Latin America have the highest number of violent cities, but violence is much more widespread. Of the 43 Latin American cities on the 2014 list, 40% have homicide rates greater than 50 per 100 000 people, and 46% have rates of 30 per 100 000 people, or more – the global average is 7 per 100 000 people. Some cities such as Juarez (Mexico), San Juan (Puerto Rico) and Medellin (Columbia) have seen sharp falls in violence, and the number of Mexican cities in the list has fallen from 25 to 2. However, the number of Brazilian cities has increased from 14 to 19, and violence has been resurgent in El Salvador after the gang truce broke down. (*insightcrime.org*, 22 Jan 2016)

➢ There are reports that Egypt’s government is increasingly using the tactic of “enforced disappearance” to crack down on real and imagined opponents. The “disappeared” are not held in the formal legal system – which had already detained thousands of people – but are moved into a network of secretive detention centers, run by the security forces. They are held without charge or access to a lawyer, where their isolation and lack of legal protection enables them to be interrogated harshly – many say they have been tortured – and forced to identify friends and relatives. Detainees are usually released without charge within months, or charged with membership of the outlawed Muslim Brotherhood. But some are missing for much longer, and the dead bodies of others are dumped in morgues. The disappeared include members of the Muslim Brotherhood, but also civil society activists, journalists and members of the public unwittingly caught up in the state’s security dragnet. “The goal seems to be to terrorise society, to show that anyone who dares criticise the government will face a similar fate,” said Mohamed Elmissiry, a researcher with Amnesty International. Public disquiet has grown over this crackdown, leading to an investigation of the cases of 101 missing people. However, lawyers and human rights groups believe that the investigation will be a whitewash, as the government has already declared that detainees were legally arrested, joined militant groups or had fled Egypt. (*Irish Times*, 27 Jan 2016)

➢ In a landmark ruling, the International Criminal Court (ICC) at The Hague has found a warlord guilty for perpetrating rape as an act of war. It also secured a conviction for “command responsibility”, which means that a commander can be found guilty of crimes if he or she orders them, even without directly taking part. This verdict was delivered against Jean–Pierre Bemba, head of the Movement for the Liberation of the Congo, who sent his militia into the Central African Republic (CAR) to rampage during a period of turmoil. More than 5200 victims testified that they had been sexually assaulted or their property stolen. Although the court has faced accusations of bias against Africa, it was an African government – the former CAR government – which referred Mr Bemba to the ICC. “The facts have shown that rape was systematic, as was pillage, and was perpetrated in a humiliating way, anywhere, anytime by multiple rapists,” said Ms Marie–Edith Douzima–Lawson, the victims’ legal representative. Sentencing has yet to be carried out. (*The Economist*, 22 Mar 2016)

➢ There is a lack of reliable data on gender violence in Cambodia, but a UN report from 2013 found that 22% of women had experienced violence from a male partner, although only 16% of men admitted violence toward a woman. In addition, 96.2% of men and 98.5% of women believe that a woman should obey her husband, and 67% of women believe they should tolerate violence to maintain the family. This attitude reflects the teachings of the Chbab Srey, a poem on women’s role which teaches submission, and which was part of the Cambodian school curriculum until 2007. It is also a legacy of the Khmer Rouge, where an unknown number of women were forced into sex work for survival, into marriage, or were victims of sexual violence. Most cases of domestic violence go unreported, partly due to Cambodia’s skeletal judicial system and lack of victim support, although tradition – which emphasizes virginity as a marriage pre–requisite – also causes victims’ silence. Rape is also prevalent in Cambodia, with 20% of men admitting to at least one rape – and 38.4% of these men were unpunished. However, the Asia Foundation has funded the development of mobile solutions by women’s networks, such as apps to explain the causes and risk factors behind domestic violence, give the names of support organisations, and to file reports anonymously. This is a small step toward making Cambodia safer for women. (*The Diplomat*, 18 Apr 2016)

## SCIENCE AND TECHNOLOGY

➢ At an expert panel moderated by the US Vice President Joe Biden at the World Economic Forum in Davos, the discussion focused on the need to collect, harness and analyze Big Data to finally find a cure for cancer. There has been a huge increase in the volume of oncological data, but researchers’ ability to use it has not kept pace. The panelists highlighted the following obstacles to fully realizing the potential of Big Data. First, medical data are not standardised across platforms, and standardising it would increase the volume of data available to scientists working on specific problems. Second, patients’ concerns over privacy must be overcome. Third, few cancer patients – only 5% – volunteer for clinical trials (despite such trials offering hope to those who are have otherwise no options left) and most of those who do are not given access to their data. Mr Biden said he is dedicating his last year of office to a “cancer moonshot”, and believes that scientists are on the cusp of a breakthrough in cancer treatment. However, he acknowledges that the Big Data challenge is a fundamental challenge, and solving it will require unprecedented co–operation between professionals across many disciplines. (*Forbes*, 26 Jan 2016)

➢ According to a study published in *The Lancet Infectious Diseases*, more than 50% of HIV–positive people who are not responding to treatment have an HIV strain which is resistant to tenofovir, a key antiretroviral drug. 60% of HIV–positive people in Africa have become tenofovir–resistant, compared to 20% in Europe. Second–line drugs are available, but these are generally more expensive and have more side effects. Resistance to tenofovir can be caused by the drug regime being incorrectly followed, or the HIV–positive person becoming infected with another, tenofovir–resistant, strain of HIV. Surveillance, treatment and monitoring of HIV patients must be improved in the wake of this development, and studies are under way to determine how HIV developed tenofovir resistance. (*Tech Times*, 29 Jan 2016)

➢ The Global Trachoma Mapping Project has recently completed a global survey of trachoma – a painful [preventable], neglected tropical disease which can cause blindness – and the scale and quality of the survey means that it could be eliminated by 2020. Ethiopia highlights the advantages of disease mapping – prior to the survey in 2012, only one district had support for tackling trachoma, but now the entire country has been mapped and there is funding and support to deliver the required interventions for the entire country. Following the survey, many areas have been enrolled to receive free antibiotics donated by Pfizer via the International Trachoma Initiative. The software app developed for the survey is also being trialed for other diseases, including schistosomiasis and guinea worm disease. (*Thomson Reuters Foundation*, 10 Feb 2016)

➢ The pharmaceutical company Sanofi has assembled a team of more than 80 experts to start pre–clinical tests of a potential zika vaccine in animals, with the first human trials likely in 2017. Other companies such as Bharat Biotech, Inovio and the US National Institutes of Health are also working on vaccines, but Sanofi has the advantage of being a major vaccine producer, and the first company to develop a vaccine for the related dengue virus. This could speed up development by several years and simplify safety, as the “backbone” of the virus is already in use. Developing a zika vaccine is potentially simpler compared to diseases like HIV, as the virus’s genetic code is more than 95% the same across samples. However, designing clinical trials could be complicated as pregnant women are often excluded until the drug’s safety is well–established, and ultimately the vaccine may be given to different age groups. (*Fortune*, 4 Mar 2016)

➢ Wei Zexi, a 21–year old student suffering from synovial sarcoma – a rare form of cancer – died after using an experimental cancer therapy he found online. He sought the treatment from a hospital that came top of his Baidu rankings. Baidu is China’s largest internet search engine, with 70% market share and more than 660 million people use its mobile search each month. Baidu has already faced criticism for selling listings to bidders without thoroughly checking their claims. Baidu marks its paid–for listings with “promote” in small text, but many argue that this does not adequately identify paid–for listings. Before he died, Wei accused the hospital of misleading him and his family over the treatment’s effectiveness, and criticized Baidu for selling medical search listings to the highest bidder. Baidu denies ranking hospitals in paid–for search results based on payment, although investigations have been launched into the hospital. The Chinese government will carry out an official inquiry into Baidu’s “search results for sale” business model, and will make the findings public. (*BBC*, 3 May 2016)

